# Visual integration in autism

**DOI:** 10.3389/fnhum.2015.00387

**Published:** 2015-07-01

**Authors:** Danielle Smith, Danielle Ropar, Harriet A. Allen

**Affiliations:** School of Psychology, University of NottinghamNottingham, UK

**Keywords:** autism, vision, integration, feedback, low-level, high-level, attention, stereopsis

## Abstract

Atypical integration is a topic of debate in the autism literature. Some theories suggest that altered perception in autism spectrum disorder (ASD) is due to a failure to integrate information from meaningful context into the final percept, whereas others suggest that integration of low-level features is impaired. Empirical research which forms the basis for these theories has failed to account for higher-level influences not inherent in the stimuli (i.e., instructions and goals) and assess integration at both lower and higher perceptual levels within the same task. Here, we describe how perceived expectations and goals of a task can modulate the processing of low-level visual input via the medial prefrontal cortex (mPFC). We then go on to illustrate how future research might assess the relative contribution of both low and high-level processes using the same paradigm. We conclude by recommending that when results appear conflicting, consideration of the relative strength of low-level input vs. feedback or high-level processes may prove helpful. Importantly, research in this area needs to more broadly consider the various influences on perception, and find better ways to assess the contributions of early and later visual processes.

Sensory abnormalities are observed in a large proportion of people with Autism Spectrum Disorder (ASD; Geschwind, 2009) and many clinical, parental, and personal reports focus on atypically intense attention to, or avoidance of, sensory information (Williams, 1998; Bogdashina, 2003; Grandin, 2006; Ben-Sasson et al., 2009). Although people with an ASD diagnosis exhibit superior performance in some tasks, such as block design and finding embedded figures (Muth et al., 2014), deficits are also shown in simple visual tasks such as orientation or motion detection (Simmons et al., 2009; Figure 1). There is considerable disagreement regarding the mechanisms underlying atypical perception, however a common theme is abnormal integration (Dakin et al., 2005; Simmons et al., 2009). When the ASD literature discusses visual integration, it is often within the reference frame of higher-level information derived from the stimulus such as its meaning or surrounding context. For instance, Plaisted et al. (1999) note that individuals with ASD do not exhibit a global interference effect of contextual information in a divided attention Navon-type task (Navon, 1977). However, even when stimuli appear devoid of meaning there may be higher-level influence in the form of personal goals or task expectations. Current perceptual theories of ASD disagree as to whether integration is affected at early (i.e., lower) or later (i.e., higher) levels of visual processing. To date, there has been little success in disentangling these experimentally. This paper will first give a brief overview of the visual pathway and then outline how current theories attempt to explain atypical visual integration in autism. We then discuss important considerations that need to be addressed in this area of research; specifically, how an individual’s goals or understanding of a task can exhibit modulatory feedback upon low-level vision through the medial prefrontal cortex (mPFC). Finally, we provide an example of how integration at both lower and higher levels could be assessed independently in the same task.

**Figure 1:**
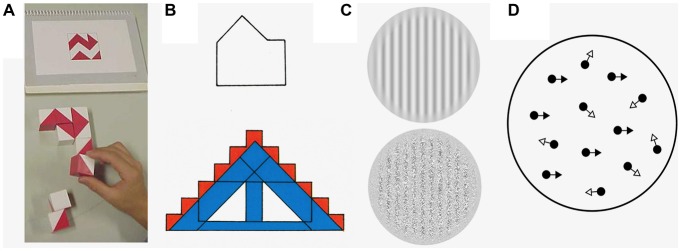
**Examples of the stimuli that are used to explore low- and high-level visual integration in autism spectrum disorder (ASD). (A)** The block design task (Wechsler, 2011). This task requires line drawings to be broken up into logical units, so that individual blocks can be used to reconstruct the original design. Here the shape above is constructed of the blocks below. **(B)**The embedded figures test (Witkin et al., 1971). Here, the participant is asked to find a simple component shape (above, in the example given) within a complex design (below). There is an increase in performance in both the block design task and the embedded figures test if there is a lack of automatic global processing. **(C)** First and second order gratings. Bertone et al. (2005) asked participants to determine the orientation of two different types of gratings, one which only contained first-order information (upper grating, which is composed of changes in luminance) and the other contained second order information (lower grating, which consists of differences in texture). **(D)** Global motion perception using random-dot kinetograms (RDKs). When presented in a motion sequence, a certain proportion of the dots in an RDK move in the same direction (signal dots; black arrows) whilst the rest move in a random direction (noise dots; white arrows)—the participant is asked to specify the perceived overall direction of the stimuli. Processing both second-order visual stimuli and RDKs involves integrating information from multiple visual channels and should be worse if there are deficits in low-level visual integration.

Visual perception is commonly conceptualized as hierarchical, with inputs arriving in area V1 from the thalamus, and being successively processed in a number of different areas. Neural response properties vary along the visual hierarchy, with latency increases that imply more complex processing when moving from earlier to later areas such as the inferotemporal cortex (Lamme and Roelfsema, 2000). Receptive field sizes also increase, implying convergence of inputs from lower- to higher-level areas (Poggio and Riesenhuber, 1999; Rao and Ballard, 1999).

Low-level integration begins to take place once simple local features, such as the orientation and location of lines and edges, are extracted from primary visual input in areas V1 and V2 (Hubel and Wiesel, 1962). The outputs of these areas, which are comprised of local representations, are then gradually consolidated, binding together different stimulus features to represent a global or overall shape at successive levels of the visual system (Kourtzi et al., 2003; Rousselet et al., 2004; Roe et al., 2012). For instance, in Figure 1B, the orientation of each line in both the simple/component and complex shapes would be extracted by early visual filters. These components would be combined at a later stage, within the lateral occipital cortex (Kourtzi and Kanwisher, 2001), to form the overall shapes.

Once simple visual features are consolidated, in feedforward models of vision, their signals are projected to higher levels of the cortex such as the inferotemporal and prefrontal areas, where sensory input is integrated with attention and task demands. Areas such as the orbitofrontal cortex and mPFC are thought to play a central role in the evaluation of potential outcomes (Shalom, 2005; Bar, 2007) by limiting the number of possible representations for a viewed object (Bar et al., 2006), assisting with identification of objects, and categories (Tanaka, 1996). Alternative conceptualizations of the visual system (e.g., Hochstein and Ahissar, 2002) propose that this higher level processing occurs early and projects information downsteam as needed, biasing early visual processing from the start.

In Figure 1B, the extraction and integration of component edges is influenced by possible representations of the overall shape (i.e., a house or a rectangle with a triangle on top). If an individual identifies the target for which they are searching as a “house” this will increase and reinforce attention towards the outline of this shape. However, integration of irrelevant distractor features, such as color, may impair the identification of the house.

## Integration in ASD

There is consistent evidence of an atypical visual processing style of ASD (Dakin and Frith, 2005; Behrmann et al., 2006; Simmons et al., 2009), commonly manifesting as deficits in global processing (i.e., processing of the whole object or scene) or superior low-level processing. Most current theories of ASD attempt to provide explanations for this atypical integration.

Weak central coherence theory (WCC; Happé and Frith, 2006) proposes that individuals with ASD have a detail-focused cognitive style where they are unable to bind details into more global forms. There is also a bias away from integrating higher-level information such as context. In support, individuals with ASD have been shown to exhibit faster performance in tasks involving embedded figures (Shah and Frith, 1983; Jolliffe and Baron-Cohen, 1997) and block design (Shah and Frith, 1993), where a lack of global feature-binding would be advantageous.

In contrast to WCC, the enhanced perceptual functioning (EPF) hypothesis (Mottron and Burack, 2001) focuses on evidence that low-level perception is enhanced in people with ASD. Mottron et al. (2006) suggest that the integration of “higher-order” information—which is automatic in typically developing/developed (TD) populations—is optional in those with ASD, meaning that the default setting of perception is more locally-oriented. Basic visual functioning may be superior in ASD populations but low-level integration of features may be impaired. This is supported by the literature that has demonstrated that although people with ASD appear to have intact or superior processing of simple dynamic stimuli (Bertone et al., 2005; Pellicano et al., 2005), they exhibit poor performance when required to combine simple visual features such as in texture-defined second-order gratings (Bertone et al., 2005) or motion coherence tasks (Milne and Szczerbinski, 2009; Koldewyn et al., 2011).

These theories conceptualize high-level and low-level processes as separate entities. However, these processes are not so easily dissociable; neurons in the visual cortex receive feedforward information (from the retina), feedback (from higher cortical areas) and have inputs from lateral connections. Research has increasingly focused on examining the interplay between these sources of information using a variety of tasks including figure-ground segregation (Vandenbroucke et al., 2009), degraded face and object recognition (Loth et al., 2010), and contextual modulation caused by collinear facilitation and contour integration (Jachim et al., 2015). All found a difference in modulatory feedback in individuals with autism. The exact nature of this difference in ASD is yet to be determined: it has been proposed to be both enhanced (Vandenbroucke et al., 2009), and reduced (Loth et al., 2010; Jachim et al., 2015).

Hypo-prior theory (HPT) has framed the perceptual atypicalities found in ASD in terms of a failure to incorporate modulatory feedback. HPT proposes that people with ASD may perceive the world differently due to attenuated priors (Pellicano and Burr, 2012). Priors encode biases towards attributes that are most likely, based on previous experience. They can improve the efficiency of neural computations by acting as constraints and reducing noise or error. Reduced priors lead to a decrease in the influence of context and prior knowledge causing superior performance in certain tasks. Like EPF, HPT predicts that people with ASD ought to rely more heavily on low-level sensory information, but does so by implicating reduced feedback. However, high-level information may come in a number of different forms (see Brock, 2012), and in its current form, HPT only considers perceptual sources of high-level information.

Similar to HPT, Lawson et al. (2014), Sinha et al. (2014) and Van de Cruys et al. (2014) propose prediction-based explanations for ASD. Here, the relative influence of prior beliefs (high-level information) compared to sensory evidence (low-level information) is controlled by the precision of predictions made by higher-level brain areas. Sensory evidence that has not been predicted is termed “prediction error”. The precision of these prediction errors is thought to determine the relative weight of high- and low-level information. Perceptual atypicalities in ASD have been proposed to occur both due to poor prediction (Sinha et al., 2014) and an over weighting of prediction errors, leading to missing of patterns and regularities (Van de Cruys et al., 2013, 2014). In Figure 1B, for instance, the integration of the larger shape has not been reliably learnt from previous experience. A further alternative explanation proposes that the pattern of impairment is due to increased precision of low-level input, leading to over-reliance on sensory signals (Friston et al., 2013; Lawson et al., 2014). Thus, there is a reduction in the weighting of higher-level information and less suppression of sensory information by prior information. In Figure 1B, for people with ASD, there might be reduced suppression of the local features by the larger shape.

In summary, with the exception of EPF, all theories discussed illustrate how a failure to incorporate feedback correctly can result in atypical visual integration. However, most empirical research in this area has considered meaning to be inherent in the stimulus—for instance, a stimulus may be immediately identifiable or similar to stored perceptual representations in memory. However, even when a stimulus appears to be absent of meaning (e.g., abstract lines), visual processing can be affected by the viewer’s own goals or their interpretation of task expectations.

## The Role of Goals and Expectations in Visual Feedback

An individual’s goals and expectations can affect attention, reduce the time taken to respond to stimuli and increase performance (Bravo and Nakayama, 1992). Attention to a particular location, or feature, can be characterized in terms of enhancement of neural responses (gain control) and suppression of neural responses outside the focus of attention (Motter, 1993; Chen et al., 2008). The enhancement of neural responses can be observed throughout the visual cortex (Motter, 1993) and the temporal lobe (Tanaka, 1996; Liu et al., 2009). The magnitude of the attentional effects throughout the visual system depends upon the nature of the task and the configuration of the stimulus (Watanabe et al., 1998; Ito and Gilbert, 1999). Li et al. (2004) trained monkeys to indicate the central point on a line and discriminate misalignment between the same lines. Critically, although identical stimuli were used in both tasks, the patterns of cell activity depended on which task was being performed. That the understanding of a task can vastly change activation patterns resonates with the effects of task instructions on cognitive tests in those with ASD (White, 2013).

Task instructions have been shown to have a differential effect on performance by those with ASD on the Navon task (Plaisted et al., 1999); when individuals were not told which level (i.e., global or local) to search for the target, those with ASD were better than typically developing (TD) children at finding the target at the local level. However, where they were told to search only one level to identify the target, both groups were faster when identifying the larger global letter than the local letter. As previously has been argued (Shalom, 2005; Bzdok et al., 2013), it is specifically under circumstances of ambiguity where those with ASD may perform differently. The mPFC may serve a specific purpose by means of establishing an order of importance (i.e., global) in TD individuals whereas it does not do so in the ASD group. When attention is directed to a single level, individuals with ASD do not perform differently as the possibilities are minimized. When cued to look for a specific feature, the selectivity of neuronal receptive fields in early visual area V1 changes to a form which approximates the cued feature (McManus et al., 2011). Even at the earliest stages of visual processing, neurons can be dynamically adjusted to be selective for complex geometries from top-down influences. Returning to Figure 1B, the failure to find the hidden figure might reflect that the entire shape is bound by a high-level automatic grouping mechanism. Once enough processing has occurred to identify the overall complex shape the filters of the early visual system can adjust to become more selective for these salient features, effectively hiding the embedded figure. It has been observed that the frontal and parietal brain areas (including the mPFC) exhibit reduced activation during the embedded figures task in ASD populations compared to controls (Lee et al., 2007; Damarla et al., 2010; Spencer et al., 2012). Previously, these areas were thought to be involved in suppression of global perceptual bias (so the reduced activation of the ASD group may be interpreted as a sign that the global form of the complex visual figure was processed to a lesser extent by the ASD than control children; Lux et al., 2004; Lee et al., 2007)—however, this can also be interpreted as reduced activation of the feedback pathways.

High-level processing is key to understanding perception and the back projections that are present throughout the visual system are likely to play an important role in the integration of low-level with high-level information. Until recently, research on visual perception in ASD has tended to focus on *either* low-level vision or higher-level influences (Simmons et al., 2009). Since differences in visual processing in individuals with ASD are not isolated to either the higher (global) or low-level (local) domains, perhaps a more parsimonious explanation is that interaction between low- and high-level mechanisms is less developed in this population. Thus, the theories that emphasize the interaction between low- and high-level mechanisms are a welcome development and are consistent with evidence that indicates that brain connectivity is disrupted in ASD (Barttfeld et al., 2011; Wass, 2011; Samson et al., 2012). Enhanced resource allocation in early visual areas, due to a lack of suppression of irrelevant details could explain the heightened performance of those with ASD in low-level visual tasks. However to-date, there is little evidence to discriminate between the specific alternatives (see Skewes et al., 2015; Westerfield et al., 2015, for two relevant studies). One paradigm that may allow differentiation between the contributions of feedforward and feedback processing in visual perception is shape constancy.

## Example: Shape Constancy

**Figure 2:**
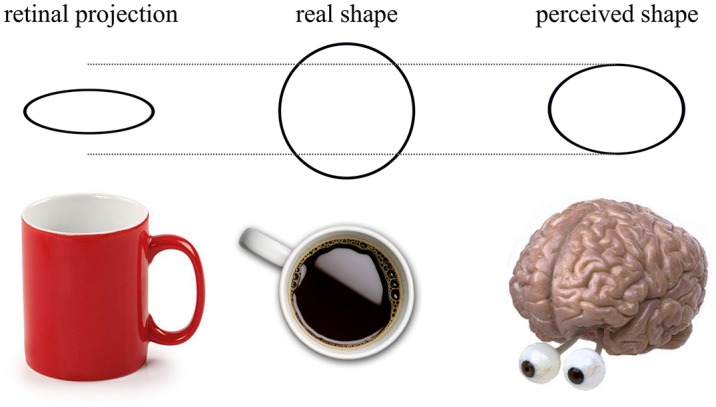
**When a cup is viewed obliquely, the retinal projection of the lip of the cup forms an ellipse**. Despite the apparent transformation, the viewer knows that the “real shape” of the lip of the cup is circular—this is the phenomenon of shape constancy. When asked to reproduce the retinal projection of the ellipse, shape constancy means that the observer is unable to accurately determine the true retinal projection. Instead, the observer will reproduce what is labeled the “perceived shape”, represented in the above image as being between the retinal projection and the real shape.

Shape constancy is the phenomenon where regardless of an object’s orientation, the shape of the object is perceived as the same (Figure 2). Ropar and Mitchell (2002) asked participants to replicate the retinal projection of a tilted circle. When participants knew the true shape, both ASD and TD participants increased the circularity of their reproduced shape, as predicted by shape constancy. However, in the ASD group this increase was significantly smaller. They concluded that perception in ASD was less influenced by prior knowledge, which can in the present context be interpreted as a reduction in the modulatory feedback. For TD participants, the higher-level information interfered with the task whereas participants with ASD reported veridical perception more easily (see, Liu et al., 2011, for a brain imaging result consistent with this interpretation).

However, shape perception and constancy can be elicited by both low- and high- level visual cues. Low-level cues to slant such as linear perspective (Howard et al., 2014) and binocular disparity (Hibbard et al., 2012) can induce shape constancy, even when the participant does not know the true shape. Ropar and Mitchell (2002) allowed binocular viewing, which may have caused shape constancy from binocular disparity. This leads to a second possible explanation of Ropar and Mitchell’s findings; individuals with ASD may be less able to utilize disparity due to an increased prevalence of deficits with convergence or strabismus (Scharre and Creedon, 1992; Milne et al., 2009). The reduction in shape constancy observed in ASD may be explained by an inability to use low-level cues to slant rather than a reduced effect of prior knowledge.

A final possibility is that feedback connections caused prior knowledge of true shape to change perception by adjusting receptive fields. It may be the case that people with ASD *were* influenced by prior knowledge but its modulatory effect was different compared to TD groups. For instance, prior knowledge may cause a decrease in shape constancy elicited by binocular disparity for participants with ASD but actually increase the efficacy of disparity for the TD group.

We aim to highlight the importance of considering perception as an interactive and dynamic process where the integration of low- and high-level sources of information may differ between populations. Research in this domain is often unable to come to a consensus due to conflicting data. Methodological differences may account for these discrepancies. For instance, some existing results appear dependent on participant characteristics such as IQ (Jarrold and Brock, 2004), on task demands (e.g., with the Navon task; Navon, 1977) or on the exact instructions given (Plaisted et al., 1998; Ropar and Mitchell, 1999, 2001). Each of these changes will produce different top-down modulations of low-level mechanisms, relating to different connectivity pathways between frontal and visual regions (for a review, see Martínez-Sanchis, 2014). Within the context of shape constancy, it has been found that results depend on how subjects interpret instructions. Even when they are instructed to replicate the retinal projection, they may believe they are being asked to replicate a shape that matches the actual inclined shape (Howard et al., 2014). Differing interpretation of instructions can change expectancies, modulating the importance and/or salience of different features of the stimulus. This will affect the strength and nature of the low-level processing and modulatory feedback. We therefore recommend that when results are conflicting and appear dependent on participant characteristics, task demands or stimulus features, considering the results in light of possible interplay between low- and high-level processes is helpful.

## Conflict of Interest Statement

The authors declare that the research was conducted in the absence of any commercial or financial relationships that could be construed as a potential conflict of interest.
